# Incidence and Risk of Hypertension in Cancer Patients Treated With Atezolizumab and Bevacizumab: A Systematic Review and Meta-Analysis

**DOI:** 10.3389/fonc.2021.726008

**Published:** 2021-10-12

**Authors:** Linhan Jiang, Xiaoxia Tan, Jun Li, Yaling Li

**Affiliations:** ^1^ Department of Pharmacy, The Affiliated Hospital of Southwest Medical University, Luzhou, China; ^2^ School of Pharmacy, Southwest Medical University, Luzhou, China; ^3^ Department of Anorectal, The Affiliated Hospital of Southwest Medical University, Luzhou, China

**Keywords:** cancer, atezolizumab, bevacizumab, drug combination, hypertension

## Abstract

**Purpose:**

This study aims to inform previous clinical assessments to better understand the total risk of hypertension with atezolizumab and bevacizumab (hereafter referred to as “A-B”) in cancer patients, and reduce future incidence of hypertension-related cardiovascular complications.

**Methods:**

Databases, including PubMed, Embase, Cochrane, and Web of Science were searched to identify relevant studies, which were retrieved from inception to March 6, 2021. Studies focused on cancer patients treated with A-B that provided data on hypertension were included. Statistical analyses were conducted to calculate hypertension incidence and relative risk (RR) with a random-effects or fixed-effects model, hinging on heterogeneity status.

**Results:**

Ten studies including 2106 patients with renal cell carcinoma (RCC), hepatocellular carcinoma (HCC), ovarian cancer, anal cancer, neuroendocrine tumors (NETs), and cervical cancer were selected for this meta-analysis. For patients treated with A-B, the all-grade and high-grade (grade 3) hypertension incidence were 31.1% (95% CI: 25.5-37.3) and 14.1% (95% CI: 10.9-18.1), respectively. No significant difference was observed in all-grade hypertension incidence between RCC and a non-RCC patients (32.9% [95% CI: 25.3-42.6] *v.s.* 29.2% [95% CI: 19.7-39.6)]). However, the number of high-grade hypertension incidence in RCC patients (9.4% [95% CI: 4.1-21.3]) was lower than that of non-RCC patients (15.6% [95% CI: 12.8-19.1]). RCC or HCC patients who received the A-B treatment were associated with significantly increased risk of all-grade hypertension with a RR of 7.22 (95% CI: 3.3-15.7; *p* = 0.6) compared with patients treated with atezolizumab.

**Conclusions:**

Cancer Patients treated with atezolizumab and bevacizumab have a significantly increased risk of hypertension. Sufficient monitoring is highly recommended to prevent the consequences of treatment-induced hypertension and other cardiovascular complications.

## Introduction

Anti-vascular endothelial growth factor (VEGF) antibodies and programmed death 1 (PD-1)/programmed death ligand 1 (PD-L1) antibodies are novel and commonly used treatments for cancers (1, 2). Atezolizumab is a monoclonal immunoglobulin G antibody that binds to and inhibits the PD-L1 (3). Bevacizumab is a recombinant, humanized monoclonal blocking antibody specific for VEGF (4). The clinical activity of atezolizumab and bevacizumab (hereafter referred to as ”A-B”) was initially innovated in a phase Ib randomized clinical trial. It was discovered that the A-B combination improves antigen-specific T-cell migration in metastatic RCC patients (5). Similarly, a phase II trial (IMmotion150) also showed improved progression-free survival in A-B-treated metastatic renal cell carcinoma (mRCC) patient expressing PD-L1 (6). A phase III clinical trial (IMmotion151) confirmed the aforementioned finding and showed a favorable safety profile (7). Additionally, A-B has clinical activity in unresectable hepatocellular carcinoma (8, 9). Currently, A-B has been approved by the United States Food and Drug Administration (FDA) for treating patients with advanced unresectable or metastatic HCC (10). In general, the purpose of the drug combination is to increase clinical efficacy and minimize drug resistance, offering a favorable therapeutic outcome. However, even though combination improves clinical efficacy, there is still uncertainty about whether the specific combination of A-B minimizes side effects.

Previously, A-B was shown to induce adverse effects including hypertension, proteinuria, fatigue, musculoskeletal pain, hyponatremia, infection, bowel obstruction, and nausea. In particular, hypertension is a major side effect, with its incidence ranging from 18.2~47.5% (11, 12). The monitoring and treatment of hypertension are therefore crucial in managing side effects in patients treated with A-B, especially in RCC patients suffering from kidney dysfunction. In a phase Ib trial (GO30140) (9), a phase II trial (IMmotion 150) (6), and two phase III trials (IMbrave 150, IMmotion 151) (7, 8), proteinuria was more frequent in the A-B groups than the control. However, there is uncertainty about whether the combination of A-B showed a higher incidence of hypertension compared with monotherapy. There have been no reports or meta-analyses of the incidence of hypertension in patients treated with A-B and the total risk of hypertension with A-B is unclear.

Given the increasing use of A-B in clinical applications, and the fact that hypertension, if not promptly recognized, can lead to major adverse cardiovascular events, we conducted a systematic review and meta-analysis to estimate the incidence and overall risk of hypertension with A-B among patients with cancer.

## Methods

This study followed the Preferred Reporting Items for Systematic reviews and Meta-Analyses (PRISMA) guidelines (13).

### Literature Search

PubMed, Embase, Cochrane, and Web of Science were searched to identify published studies on the incidence and risk of hypertension in cancer patients treated with A+B. Studies were retrieved from inception to March 6, 2021, and no language restrictions or publication starting date limitations were applied. The search terms included atezolizumab, MPDL3280A, Tecentriq, Bevacizumab, Mvasi, Avastin, Neoplasms, Tumors, Hypertension, and related free words (**Supplementary Material 1**).

### Selection Criteria

All published clinical trials and observational studies were included. Conference abstracts, reviews, individual cases, editorials, letters to the editor and publishers, concerning non-human studies, and other literature with unavailable study data were excluded. Relevant data were extracted independently by two reviewers, with differences reconciled by the third reviewer.

### Data Extraction

Two reviewers performed independent double data extraction. The following information was obtained from each study: first author’s name, year of publication, region, study design, trial phase, number of arms, treatment arms, number of patients enrolled, number of events or incidences of hypertension, median age, underlying malignancy (**Table 1**). The incidence of hypertension was calculated for the cumulative incidence. All-grade and high-grade (grade 3) hypertension, as defined by 2018 ESC/ESH Clinical Practice Guidelines for the Management of Arterial Hypertension (17), were included in the analysis.

**Table 1:** Characteristics of the trials and patients included in the meta-analysis.

Author	Year	Region	Study Design	Phase	Arm	Treatment arms		Patients enrolled, n	Sample size	Sex		Median age, years	malignancy
										Women	Men		
Moroney et al. (14)	2020	USA	Single arm	Ib	1	A-B		20	20	20 (100%)	0 (0%)	59 (37-80)	OC
Morris et al. (15)	2020	USA	Single arm	II	1	A-B		20	20	–	–	59 (43-80)	AC
McGregor et al. (16)	2020	USA	Single arm	II	1	A-B		60	60	13 (22%)	47 (78%)	61 (22–82)	RCC
Lee et al. (9)	2020	USA, AUS, China, Japan, Korea, New Zealand, Taiwan	RCT	Ib	2	A-B Atezolizumab		223	104	20 (19%)	84 (81%)	62 (23–82)	HCC
Halperin et al. (12)	2020	USA	Single arm	II	1	A-B		40	40	–	–	–	NETs
Friedman et al. (11)	2020	USA	Single arm	II	1	A-B		11	11	11 (100%)	0 (0%)	48 (31–55)	CC
Finn et al. (8)	2020	USA, AUS, Canada, China, Czechia, France, Germany, Hong Kong, Italy, Japan, Korea, Poland, Russia, Singapore, Spain, Taiwan, UK	RCT	III	2	A-B Sorafenib		501	336	109 (18%)	227 (82%)	64 (56–71)	HCC
Rini et al. (7)	2019	USA, AUS, Bosnia and Herzegovina, Brazil, Canada, Czechia, Denmark, France, Germany, Italy, Japan, Korea, Mexico, Poland, Russia, Singapore, Spain, Taiwan, Thailand, Turkey, UK	RCT	III	2	A-B Sunitinib		915	454	137 (30%)	317 (70%)	62 (56–69)	RCC
McDermott et al. (6)	2018	USA, Czechia, France, Germany, Italy, Poland, Romania, Spain, UK	RCT	II	3	A-B Atezolizumab Sunitinib		305	101	27 (27%)	74 (73%)	62 (32-88)	RCC
Wallin et al. (5)	2016	USA	Single arm	Ib	1		A-B	11	10	3 (27%)	8 (73%)	59 (42-74)	RCC

RCT, randomized controlled trial; A-B, atezolizumab, and bevacizumab; -, not available; OC, ovarian cancer; AC, anal cancer; RCC, renal cancer carcinoma; HCC, hepatocellular carcinoma; NETs, neuroendocrine tumors; CV, cervical cancer.

### Statistical Analysis

#### Qualitative Synthesis

The characteristics and quality of the included studies were assessed by two reviewers using the Newcastle-Ottawa scale (NOS) independently (18). Disagreements were resolved by discussion and further review.

#### Quantitative Synthesis

For this meta-analysis, both the fixed-effects and random-effects models were considered, hinging on the heterogeneity across included studies (19, 20). Significant heterogeneity was identified to exist when *p *< 0.1 or I^2 ^> 50% (21).

#### Sources of Bias

Publication bias was evaluated by visual inspection of funnel plots and with both the Begg’s and Egger’s tests.

#### Heterogeneity Analysis

The *Q* tests and I^2^ index were estimated to quantified heterogeneity.

#### Statistical Software

All statistical analyses were performed with R software (version 4.0.2). A *p* value ≤ 0.05 was considered significant.

## Results

### Data Extraction And Quality Assessment

#### Systematic Review Process

**Figure 1:**
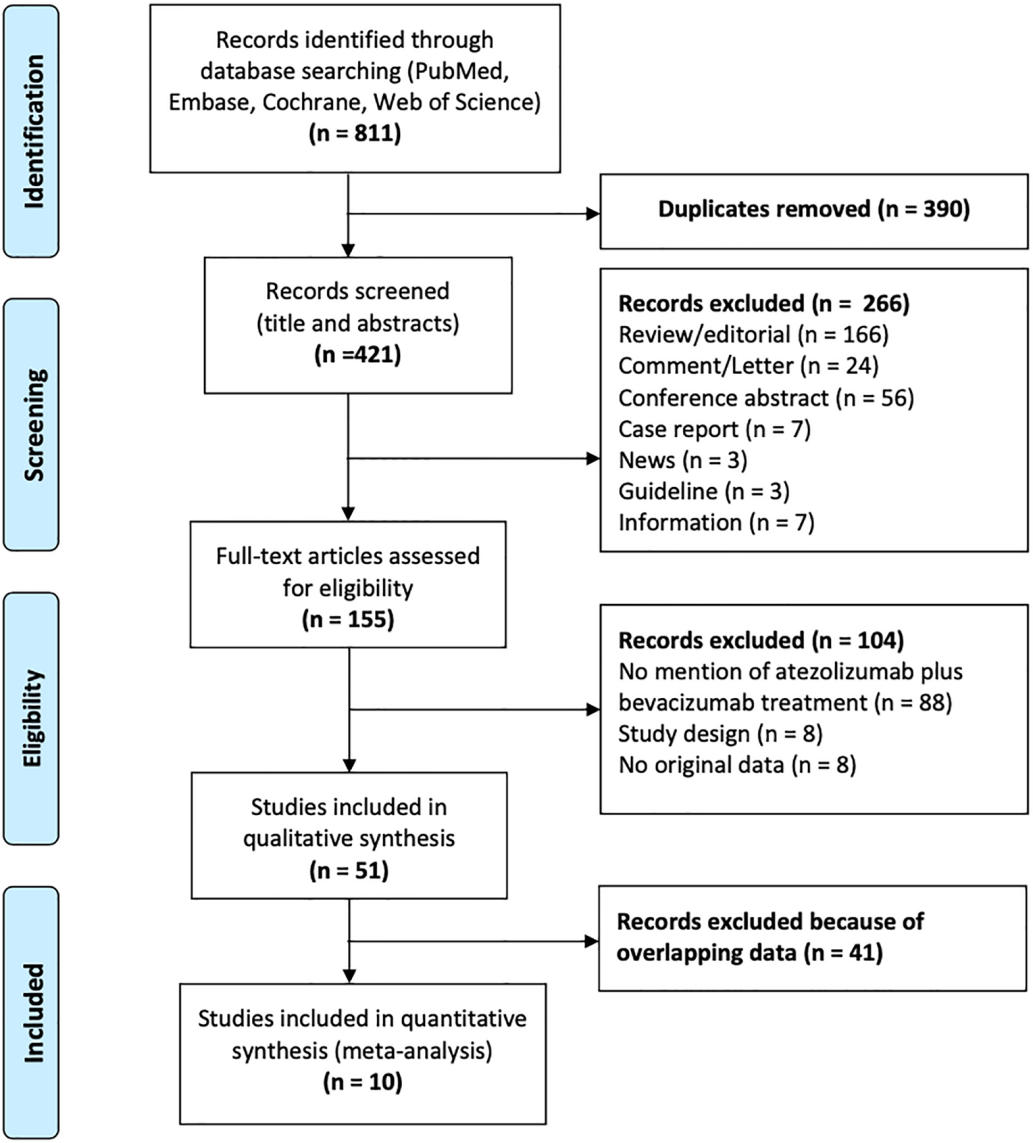
Flow chart of the selection of publications included in the meta-analysis.

A total of 811 studies were identified, of which 390 were removed owing to duplication or overlap (determined using Endnote software). Another 266 studies were excluded with screening titles and abstracts. Out of the remaining 155 full-text studies, 104 were excluded. Ultimately, 10 studies were eligible for analysis. **Figure 1** shows a flow chart depicting the process of publication selection.

#### Quality Assessment With the Newcastle-Ottawa Scale

To evaluate the quality of the evidence, the Newcastle-Ottawa quality assessment scale was used. According to the Newcastle-Ottawa Scale, all selected studies achieved at least 6 stars, indicating a low to moderate risk of bias (**Supplementary Table 1**).

#### Characteristics of Eligible Studies

In total, 2106 patients were eligible for analysis, and 1156 patients were treated with A-B, versus 950 patients treated with other treatments included atezolizumab, sorafenib, and sunitinib. The characteristics of the included studies are shown in **Table 1**. All studies were interventional clinical trials, with 10 studies including 4 randomized controlled trials and 6 single-arm trials. There was no mention of preexisting hypertension in all trials. The underlying malignancies included RCC, HCC, ovarian cancer, anal cancer, neuroendocrine tumors (NETs), and cervical cancer. The dose and schedule of A-B was 1200 mg of atezolizumab plus 15 mg per kilogram of body weight of bevacizumab intravenously every 3 weeks in all trials.

### Evidence Synthesis

#### The Overall Incidence of Hypertension

**Figure 2:**
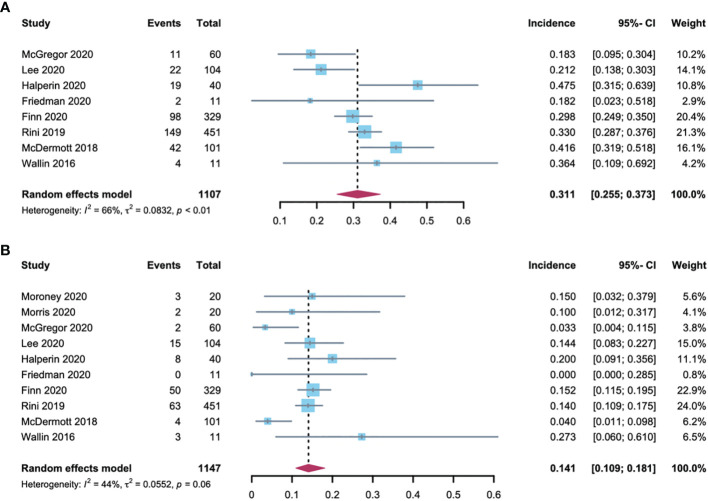
Forest plot of hypertension incidence in cancer patients treated with **(A, B)**. **(A)** incidences of all-grade hypertension; **(B)** incidences of high-grade hypertension.

Data regarding all-grade hypertension were available for analysis from 8 trials including 1107 patients who had various tumors and received A-B. The all-grade hypertension incidence ranged from 18.2% to 47.5%, with the lowest incidence noted in an advanced cervical cancer clinical trial (11), and the highest in an advanced neuroendocrine tumor clinical trial (12). The heterogeneity statistic showed significance across the studies included in the meta-analysis (*I^2^* = 66%, *p* < 0·01). We performed a sensitivity analysis conducted in which any single study was excluded by turn to explore the source of heterogeneity. The results did not change significantly, proposing the robustness of these findings (**Supplementary Figure 1**). As analyzed by a random-effects model, the all-grade hypertension incidence in patients treated with A-B was 31.1% (95% CI: 25.5-37.3; **Figure 2A**).

High-grade hypertension cannot be controlled with monotherapy that otherwise leads to life-threatening consequences, resulting in adverse effects or even A-B discontinuation. Data regarding high-grade hypertension were assessable for analysis from 10 trials, which included 1147 patients. The grade 3 hypertension incidence ranged between 0% and 27.3%, with the lowest in the advanced cervical cancer trial (11), and highest in the metastatic renal cell carcinoma trial (5). The heterogeneity of the included studies was *I^2^* = 44% (*p* < 0·01). A sensitivity analysis was performed to explore the source of heterogeneity and the results were robust (**Supplementary Figure 1**). As analyzed by the random-effects model, the summary estimate for the incidence of high-grade hypertension was 14.1% (95% CI: 10.9-18.1; **Figure 2B**).

#### Incidence of Hypertension in RCC and Non-RCC

**Figure 3:**
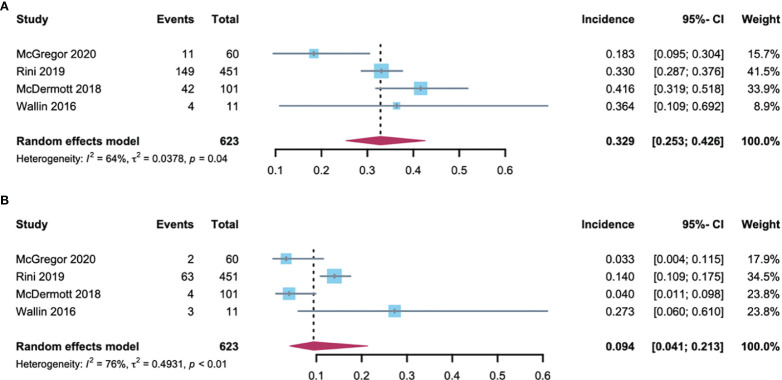
Forest plot of the A-B-associated hypertension incidence in RCC patients. **(A)** incidences of all-grade hypertension with RCC; **(B)** incidences of high-grade hypertension with RCC.

**Figure 4:**
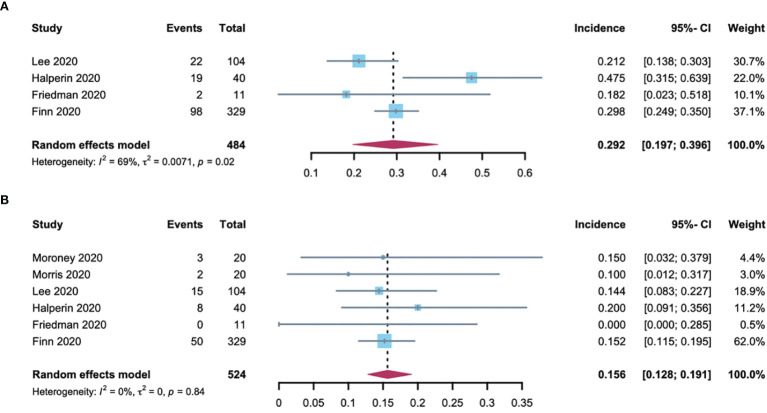
Forest plot of the A-B-associated hypertension incidence in non-RCC patients. **(A)** incidences of all-grade hypertension with non-RCC; **(B)** incidences of high-grade hypertension with non-RCC.

RCC patients are more burdened by hypertension due to previous renal parenchymal disease and renal insufficiency and sufficient evidence demonstrating that hypertension predisposes them to renal cell cancer development (22). In addition, among the 2106 patients included in the analysis, 61% had RCC. As such, further analysis of the hypertension incidence in RCC patients compared with non-RCC patients is required. Using the random-effects model (considerable heterogeneity, *I^2^* = 64%, *p* = 0·04; *I^2^* = 76%, *p* < 0·01), the incidence of all-grade and high-grade hypertension was 32.9% (95% CI: 25.3-42.6) and 9.4% (95% CI: 4.1-21.3) respectively in RCC patients (**Figure 3**). The all-grade and high-grade hypertension incidence were 29.2% (95% CI: 19.7-39.6) and 15.6% (95% CI: 12.8-19.1) respectively in non-RCC patients, as determined by the random-effects model (considerable heterogeneity, *I^2^* = 69%, *p* = 0.02) in all-grade and fixed-effects model (no heterogeneity, *I^2^* = 0%, *p* =0.84) in high-grade hypertension (**Figure 4**). All results did not significantly change by sensitivity analysis, hence proposing the robustness of these findings (**Supplementary Figure 1**). No significant difference was detected in the all-grade hypertension incidence (RR 1.14[95% CI 0.95-1.36]) and high-grade hypertension incidence (RR 0.78 [95% CI 0.58-1.05]) between RCC and non-RCC patients.

#### RR of Hypertension

**Figure 5:**
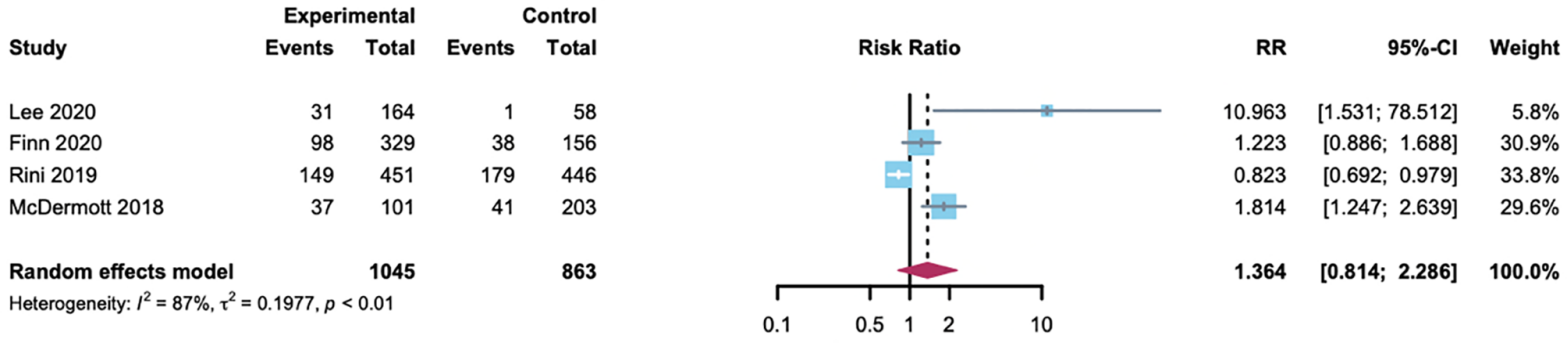
The RR of atezolizumab plus bevacizumab-associated hypertension versus control.

The summary RR for hypertension with A-B compared with the control group in HCC or RCC patients was done among the 1908 patients from four randomized controlled trials. Two trials used atezolizumab as the control, and the other used sorafenib or sunitinib. In addition to a trial for RCC (40.1%) (7), hypertension with lower incidence in the control group (1.7%, 20.2% and 24.4%, respectively) (6, 8, 9). In conclusion, no evidence was found of an association between A-B and a significantly increased risk of hypertension compared with control (RR 1.36 [95% CI 0.81-2.29]; **Figure 5**). The heterogeneity statistic showed significance across the studies included in the meta-analysis (*I^2^* = 87%, *p* < 0·01). Thus, we performed a sensitivity analysis and the results proposed the robustness of these findings (**Supplementary Figure 1**). To better understand the possible reasons for the heterogeneity, we then performed subgroup analyses by drug type. In the atezolizumab subset, the incidence of hypertension was statistically higher among patients on A-B therapy (RR 7.22 [95% CI 3.3-15.7]; **Supplementary Figure 2**). As a consequence, A-B was associated with a significantly increased risk of hypertension in patients compared with atezolizumab.

#### Publication Bias

No significant publication bias was indicated for all-grade hypertension and high-grade hypertension by either the Egger’s test (*p* = 0.72 and 0.40, respectively) or the Begg’s test (*p* = 0.46 and 0.33, respectively). The funnel plots, Egger’s test, and Begg’s test are provided in **Supplementary Figures 3**–**5**.

## Discussion

In 2019, more than 16.9 million Americans with a history of cancer were alive, and this number is projected to grow to more than 22.1 million by 2030 (23). The development of novel anticancer drugs has significantly contributed to increased survival rates for patients with cancer over recent decades and comes at the cost of potential short-term and long-term toxicities (24). Cardiovascular toxicity is nonnegligible and adversely affects outcomes (25). Hypertension is an important risk factor for cardiovascular diseases and a serious global problem. The prevalence of hypertension has doubled in the past three decades, accounting for 8.5 million deaths annually worldwide (26, 27). More importantly, Hypertension was the most common comorbidity among patients with cancer in a large observational cohort study, with a reported prevalence of 38% (28). As understanding of targeted therapies improves, there is growing awareness of the importance and detrimental vascular effects of a new generation of antitumor agents (29, 30). In this study, we performed a meta-analysis of the incidence and risk of hypertension in cancer patients treated with A-B. These findings have important clinical implications for quantifying the risks of hypertension in considering the trade-off of A-B treatment during shared decision-making.

Solid tumors are angiogenesis-dependent for growth and metastases. Currently, several proangiogenic factors have been identified, among which VEGF is a critical mediator that promotes angiogenesis (31). Anti-VEGF therapy was demonstrated a significant antitumor effect, leading to the rapid development of the VEGF signaling pathway (VSP) inhibitors, which are an approved treatment of a broad spectrum of malignancies. However, wide clinical application of VSP inhibitors is accompanied by increasing incidence of cardiovascular risk, and increasing hypertension, arterial or venous thrombotic events, and heart failure (32). Hypertension associated with anti-programmed death-1 agents and angiogenesis inhibitors is an issue that cannot be ignored in patients receiving A-B therapy. In addition to atezolizumab, several other anti-programmed death-1 agents, such as durvalumab and avelumab appear to correlate with the genesis of hypertension (**Table 2**). Likewise, apart from bevacizumab, other angiogenesis inhibitors such as sorafenib, sunitinib, cediranib, ramucirumab, and apatinib are also associated with hypertension (**Table 3**). In a study by Abdel-Qadir and colleagues, angiogenesis inhibitors were demonstrated to be corrected with hypertension incidence of 22.1% and an OR of 5.28 [95%CI: 4.53–6.15] (40). Our study demonstrates that A-B appears to correlate with a significantly increased risk of hypertension incidence of 31.1% (95% CI: 25.5-37.3) and an RR of 7.22 (95% CI: 3.3-15.7; *p* = 0.6) compared with atezolizumab in a subset that included patients with RCC or HCC. Thus, when considered these findings together, A-B was significantly associated with a considerable risk of hypertension in RCC, HCC, ovarian cancer, anal cancer, NETs, and cervical cancer.

**Table 2:** Hypertension risk with programmed death ligand 1 (PD-L1) antibodies.

	Molecular target	Incidence of hypertension	Relative risk of hypertension	Reference
Atezolizumab	PD-L1	0%-19%	–	(9, 33–35)

PD-L1, programmed death ligand 1; -, not available.

*durvalumab plus ramucirumab, durvalumab plus olaparib/cediranib, and velumab plus axitinib associated with hypertension.

**Table 3:** Hypertension risk with angiogenesis inhibitors.

	Molecular target	Incidence of hypertension	Relative risk of hypertension	Reference
Bevacizumab	Anti-VEGF-A antibody	25.4% (21.3–30.1)	7.5 (4.2-13.4)	(36)
Sorafenib	B-Raf, FLT-1, FLT-3, FLT-4, KDR, KIT, PDGFR-A, PDGFR-B, FGFR, c-fms	22.5% (19.5–25.9)	3.9 (2.6-5.9)	(37)
Sunitinib	ABL-1, c-KIT, PDGFR-A, PDGFR-B, FLT-1, KDR, FLT-3, FLT-4, FGFR, SRC, c-smc	29%	–	(38)
Ramucirumab	Anti-KDR antibody	21.3%	2.7 (2.3-3.2)	(39)

VEGF, anti-vascular endothelial growth factor; -, not available; VEGFR, vascular endothelial growth factor receptor; PDGFR, platelet-derived growth factor receptor.

At present, the mechanisms of hypertension have not been fully understood. However, many possible mechanisms have been proposed. One of the key mechanisms by which VEGF signaling pathway inhibitors mediate hypertension is through acute inhibition of endothelial-derived vasodilatory factors such as nitric oxide (NO) (41). Activation of VEGF induces rapid hypotension, through upregulating NO synthase by PI3k/Akt and MAPK dependent pathways in endothelial cells, which promotes NO production, vascular permeability, and vascular vasodilation. Treatment with VSP inhibitors has been demonstrated to decrease NO synthesis and lead to hypertension (42). Other mechanisms include rarefaction, a process of impaired angiogenesis in normal, nontumor tissue, and neurohormonal activation, or the renin angiotensin aldosterone system (RASS), which likely play roles (43). The association of A-B with hypertension might be directly correlated to the inhibition of bevacizumab on the vascular endothelial growth factor receptor (VEGFR).

Hypertension was independently related to RCC risk, which can either be an independent risk factor for RCC as a result of chronic renal hypoxia and angiogenesis or RCC patients might have an increased risk of hypertension due to previous renal parenchymal disease and renal dysfunction (44, 45). Our study showed that the all-grade hypertension incidence in RCC patients (32.9% [95% CI: 25.3-42.6]) is higher than non-RCC patients (29.2% [95% CI: 19.7-39.6]) when they are treated with A-B; however, the high-grade hypertension incidence in RCC patients (9.4% [95% CI: 4.1-21.3]) is lower than those with non-RCC (15.6% [95% CI: 12.8-19.1]). A possible explanation that might account for these findings is that the A-B has elevated blood pressure and induced hypertension prominently so that the difference between RCC and non-RCC becomes unapparent. It is upheld by the observation that a high incidence in all-grade hypertension with A-B (31.1% [95% CI: 25.5-37.3]) was noted in this study. Another explanation is that A-B are mainly metabolized by the liver (46, 47). Previous nephrectomy and RCC-related renal insufficiency might not have a substantial potential effect on the concentration of A-B.

Hypertension is mainly caused by bevacizumab and several opinion managements of bevacizumab-associated hypertension applied on A-B treatment perhaps are available. According to the drug label information for bevacizumab, there is a higher incidence of severe hypertension in patients who received bevacizumab compared with those who received chemotherapy. Across clinical studies, with high-grade hypertension incidence was 5~18%, and monitoring of blood pressure is needed biweekly or every 3 weeks during the treatment of A-B (46). Thereafter, hypertension should be treated with appropriate antihypertensive therapy and regular monitoring. Blood pressure should also be monitored regularly in patients with bevacizumab-induced or -exacerbated hypertension after discontinuing. Discontinuation in cases of patients not fully controlled by medication, hypertensive crisis, or hypertensive encephalopathy (46). According to this study, all-grade hypertension incidence is (31.1% [95% CI: 25.5-37.3]) and high-grade hypertension is 14.1% (10.9-18.1), and perhaps similar monitoring, control, and treatment deserve consideration. Based on the recommendations of the European Society of Cardiology (ESC), angiotensin converting enzyme inhibitors (ACEI), angiotensin receptor blockers (ARBs), and dihydropyridine calcium channel blockers (CCBs) have been recommended as first-line treatments (43). Otherwise, beta-blockers may also be taken into account due to their effects on NO and vasodilation. Additionally, salt restriction might prevent VEGF inhibitor-induced toxicity since VEGF inhibitor-induced hypertension is salt-sensitive (48).

Drug interactions are also a significant concern for cancer patients. In a previous study, after 4 cycles of therapy (at Day 63), 3 out of 8 patients who received bevacizumab with paclitaxel and carboplatin had lower paclitaxel exposure than initiation (Day 0). In comparison, patients who received paclitaxel and carboplatin had a better paclitaxel exposure at Day 63 compared with Day 0. Fortunately, the current study shows that no potential drug-drug interactions are presented in atezolizumab. Likewise, there are no interactions of bevacizumab in other drugs. When bevacizumab was administered in combination with irinotecan or SN38, interferon-α, carboplatin, or paclitaxel, no clinically significant interaction on the pharmacokinetics was observed (46).

Many risk factors may also have additive effects on hypertension in patients with cancer. As the most frequent comorbidity among patients with cancer, hypertension has a relatively high proportion among those with preexisting hypertension, older age, and high body mass index (28). Several agents have also been shown to induce or aggravate previously controlled hypertension. As discussed previously, VEGF is the drug class that exhibits the strongest association with hypertension in most relevant randomized trials (49). Other anticancer therapies linked to hypertension include cisplatin derivatives, proteasome inhibitors, corticosteroids, alkylating agents, interferon-alpha, radiation therapy, inhibitors of the mammalian target of rapamycin (mTOR inhibitors), taxanes, vinca rosea alkaloids, and gemcitabine (50). Nonantineoplastic agents, on the other hand, include immunosuppressive agents (cyclosporine, tacrolimus), erythropoietin, and nonsteroidal anti-inflammatory drugs (NSAIDs) (50). In terms of disease, aside from renal cell carcinoma, which may cause hypertension bidirectionally (51), hypertension is evident among patients with hepatocellular carcinoma and paraneoplastic syndrome (52). In addition, it is worth noting that hypertension may be due to white-coat hypertension or a reactive anxiety disorder. It is of great importance to investigate the patient’s history, and undertake ECG and echocardiography or Holter ambulatory BP monitoring (ABPM), to avoid prescribing drugs were not necessary (53).

The strengths of the study include that it represents the first meta-analysis of cancer patients treated with A-B, including 2106 patients, with 1156 patients assigned A-B. Furthermore, most of the included trials are multinational and multicentric. In addition, we sought to explore the sources of heterogeneity observed in studies through subgroup analysis by comparing different drug types. In addition, the statistical test showed no indication of potential publication bias based on our confirmation, as we attempted to diminish bias by contacting corresponding authors.

Several limitations deserve comment in our systematic review and meta-analysis. Firstly, like any other meta-analysis, our study is affected by the limitations of the included clinical trials. These trials might have underestimated A-B-associated hypertension incidence due to the imperfection of a study by Morris and colleagues (15) recording adverse events. Additionally, patients with significant cardiovascular disease, inadequately controlled hypertension, prior history of hypertensive crisis or hypertensive encephalopathy, and other cardiovascular-related abnormalities have been excluded from some studies. Therefore, the capacity for determining the overall incidence of hypertension is limited. In contrast, the baseline of hypertension was not mentioned in the included clinical trials. The omission may have contributed to an overestimation of hypertension incidence with A-B. Secondly, the cancer patients in our study were all screened from randomized clinical trials. Therefore, our findings were concluded largely from academic centers and research institutes and might not be representative of community-treated cancer patients. Thirdly, although we have concluded that there is no significant difference in hypertension incidence between RCC and non-RCC patients treated with A-B, our finding might be restricted to a small sample size of high-grade hypertension patients. Finally, the included studies showed heterogeneity in study design, population demographics, duration of follow-up, and measurement and adjustment for confounders. Despite the use of appropriate random-effect models and subgroup analyses, these differences are not explained.

This study demonstrated that the drug combination does not only not reduce side effects, it also causes more adverse reactions, and the combination of A-B is associated with an increased risk of hypertension. Sufficient monitoring and earlier administration could be considered as ways to prevent the consequences of treatment-induced hypertension and other cardiovascular complications. Further trials of A-B will be needed due to the limitations of our study, and more surveillance and reporting of the hypertensive and cardiovascular events are required to identify the individual and optimal therapeutic approach of hypertension with A-B.

## Data Availability Statement

The original contributions presented in the study are included in the article/**Supplementary Material**. Further inquiries can be directed to the corresponding authors.

## Author Contributions

LJ, XT, JL, and YL designed the study. LJ and XT identified eligible studies, assessed quality of the included studies, extracted data and performed data analyses. LJ, XT, JL, and YL wrote and revised the manuscript. All authors contributed to the article and approved the submitted version.

## Funding

This work was supported by the National Natural Science Foundation of China (81803019).

## Conflict of Interest

The authors declare that the research was conducted in the absence of any commercial or financial relationships that could be construed as a potential conflict of interest.

## Publisher’s Note

All claims expressed in this article are solely those of the authors and do not necessarily represent those of their affiliated organizations, or those of the publisher, the editors and the reviewers. Any product that may be evaluated in this article, or claim that may be made by its manufacturer, is not guaranteed or endorsed by the publisher.
