# Supplementary material for: Incidence and Risk of Hypertension in Cancer Patients Treated With Atezolizumab and Bevacizumab: A Systematic Review and Meta-Analysis
Source: Front Oncol. 2021 Oct 12;11:726008. doi: 10.3389/fonc.2021.726008 (PMC8546244; doi:10.3389/fonc.2021.726008)

Supplementary material

Contents

1. **Supplementary 1** Search strategy
2. **Supplementary table 1.** Risk of bias assessment for included trials
3. **Supplementary figure 1.** Results of sensitivity analysis
4. **Supplementary figure 2.** Forest plot for subgroup analyses in relative risk of atezolizumab plus bevacizumab-associated hypertension versus control
5. **Supplementary figure 3.** Funnel plot for publication bias
6. **Supplementary figure 4.** Assessment of publication bias by Egger's test.
7. **Supplementary figure 5.** Assessment of publication bias by Begg's test.

Supplementary 1: literature search strategy for the databases

This appendix contains listings of search terms and the number of search results used in the literature searches to develop the systematic literature review.

Search Strategy 1

Search strategy for PubMed from inception to 6th, March, 2021

#1 (atezolizumab) OR (anti-PDL1) OR (MPDL3280A) OR (MPDL-3280A) OR (Tecentriq) OR (RG7446) OR (RG-7446)

#2 (Bevacizumab) OR (Mvasi) OR (Bevacizumab-awwb) OR (Bevacizumab awwb) OR (Avastin)

#3 (Neoplasms[MeSH Terms]) OR (Neoplasia[Title/Abstract]) OR (Neoplasias[Title/Abstract]) OR (Neoplasm[Title/Abstract]) OR (Tumors[Title/Abstract]) OR (Tumor[Title/Abstract]) OR (Cancer[Title/Abstract]) OR (Cancers[Title/Abstract]) OR (Malignancy[Title/Abstract]) OR (Malignancies[Title/Abstract]) OR (Malignant Neoplasms[Title/Abstract]) OR (Malignant Neoplasm[Title/Abstract]) OR (Neoplasm, Malignant[Title/Abstract]) OR (Neoplasms, Malignant[Title/Abstract]) OR (Benign Neoplasms[Title/Abstract]) OR (Neoplasms, Benign[Title/Abstract]) OR (Benign Neoplasm[Title/Abstract]) OR (Neoplasm, Benign[Title/Abstract])

#4 (Hypertension [MeSH Terms]) OR (Blood Pressure, High[Text Word]) OR (Blood Pressures, High[Text Word]) OR (High Blood Pressure[Text Word]) OR (High Blood Pressures[Text Word])

#5 ((((((((epidemiologic studies[MeSH Terms])) OR (cohort studies[MeSH Terms])) OR (epidemiologic[Text Word])) OR (longitudinal[Text Word])) OR (cohort[Text Word])) OR (follow up[Text Word])) OR (observational[Text Word])) OR (prospective[Text Word])

#6 humans[MeSH Terms]

#7 review[Publication Type]

#8 #1 AND #2 AND #3 AND #4 AND #5 AND #6 NOT #7

Search Strategy 2

Search strategy for Embase from inception to 6th, March, 2021

#1 'atezolizumab':ti,ab,kw OR 'anti-PDL1':ti,ab,kw OR 'MPDL3280A':ti,ab,kw OR 'MPDL-3280A':ti,ab,kw OR 'Tecentriq':ti,ab,kw OR 'RG7446':ti,ab,kw OR 'RG-7446':ti,ab,kw

#2 'Bevacizumab':ti,ab,kw OR 'Mvasi':ti,ab,kw OR 'Bevacizumab-awwb':ti,ab,kw OR 'Bevacizumab awwb':ti,ab,kw OR 'Avastin':ti,ab,kw

#3 'Neoplasms':ti,ab,kw OR 'Neoplasia':ti,ab,kw OR 'Neoplasias':ti,ab,kw OR 'Neoplasm':ti,ab,kw OR 'Tumors':ti,ab,kw OR 'Tumor':ti,ab,kw OR 'Cancer':ti,ab,kw OR 'Cancers':ti,ab,kw OR 'Malignancy':ti,ab,kw OR 'Malignancies':ti,ab,kw OR 'Malignant Neoplasms':ti,ab,kw OR 'Malignant Neoplasm':ti,ab,kw OR 'Neoplasm, Malignant':ti,ab,kw OR 'Neoplasms, Malignant':ti,ab,kw OR 'Benign Neoplasms':ti,ab,kw OR 'Neoplasms, Benign':ti,ab,kw OR 'Benign Neoplasm':ti,ab,kw OR 'Neoplasm, Benign':ti,ab,kw

#4 #1 AND #2 AND #3 AND [humans]/lim

Search Strategy 3

Search strategy for Web of science from inception to 6th, March, 2021

#1 TS=((atezolizumab) OR (anti-PDL1) OR (MPDL3280A) OR (MPDL-3280A) OR (Tecentriq) OR (RG7446) OR (RG-7446))

#2 TS=((Bevacizumab) OR (Mvasi) OR (Bevacizumab-awwb) OR (Bevacizumab awwb) OR (Avastin))

#3

TS=((Neoplasms) OR (Neoplasia) OR (Neoplasias) OR (Neoplasm) OR (Tumors) OR (Tumo r) OR (Cancer) OR (Cancers) OR (Malignancy) OR (Malignancies) OR (Malignant Neoplasms) OR (Malignant Neoplasm) OR (Neoplasm, Malignant) OR (Neoplasms, Malignant) OR (Benign Neoplasms) OR (Neoplasms, Benign) OR (Benign Neoplasm) OR (Neoplasm, Benign))

#4 TS=((cohort) OR (longitudinal) OR (follow) OR (prospective) OR (observational))

#5 #1 AND #2 AND #3 AND #4

Supplementary table 1. Newcastle-Ottawa Scale for risk of bias assessment of studies included in the meta-analysis.

Study	Selection				Comparability	Outcome			Overall
	Representativeness of exposed cohort	Selection of nonexposed	Ascertainment of exposure	Outcome not present at start		Assessment of outcome	Adequate follow-up length	Adequacy of follow-up	
Moroney et al.	★	★	★	★	★	★	★	★	8
Morris et al.	★	★	★	★	☆	★	★	★	7
McGregor et al.	★	★	★	★	★	★	★	★	8
Lee et al.	★	★	★	★	★	★	★	★	8
Halperin et al.	★	★	★	★	☆	★	★	★	7
Friedman et al.	★	★	★	★	☆	★	★	★	7
Finn et al.	★	★	★	★	★★	★	★	★	9
Rini et al.	★	★	★	★	★★	★	★	★	9
Mcdermoot et al.	★	★	★	★	★★	★	★	★	9
Wallin et al.	★	★	★	★	★	★	☆	☆	6

Supplementary figure 1. Results of sensitivity analysis. A. incidences of all-grade hypertension; B. incidences of high-grade hypertension; C. incidences of all-grade hypertension with RCC; D. incidences of high-grade hypertension with RCC; E. incidences of all-grade hypertension with non-RCC; F. incidences of high-grade hypertension with non-RCC; G. relative risk of atezolizumab plus bevacizumab-associated hypertension versus control from the four randomized controlled trials of patients.

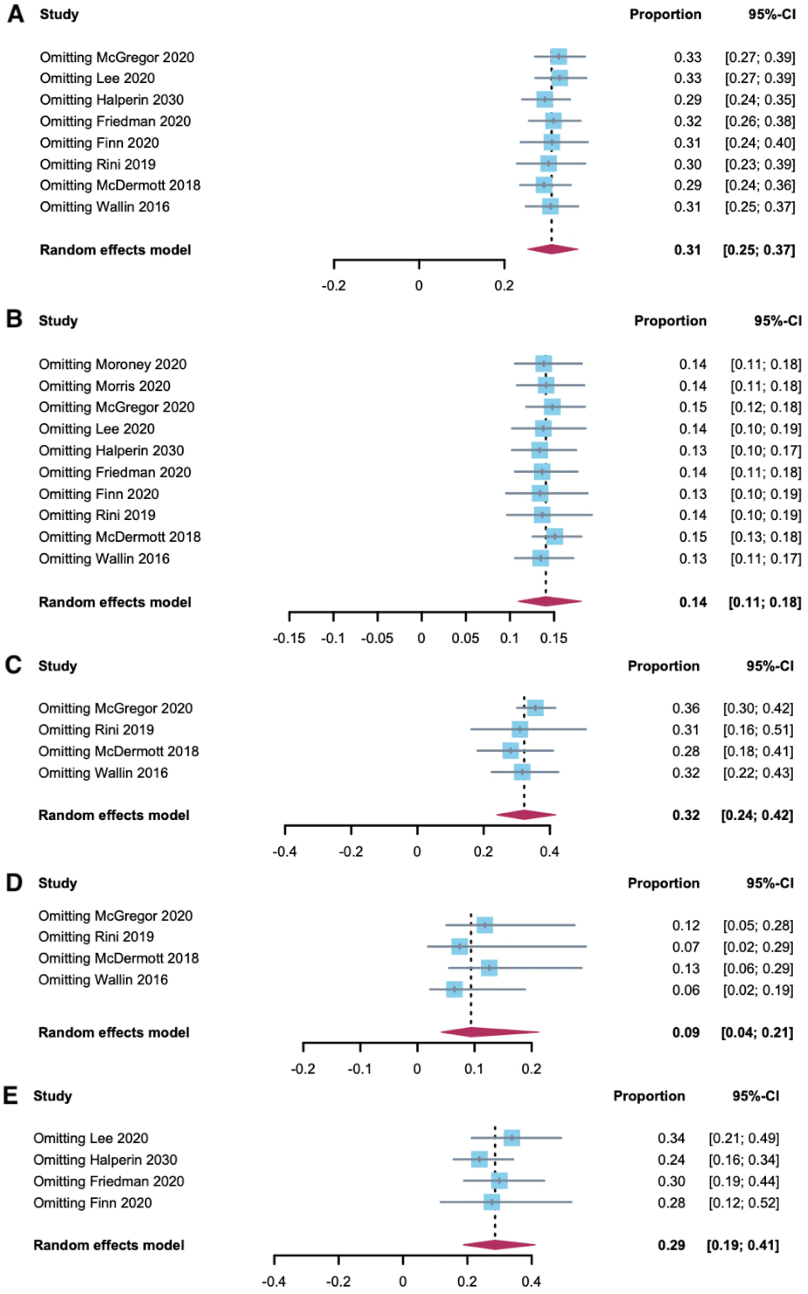

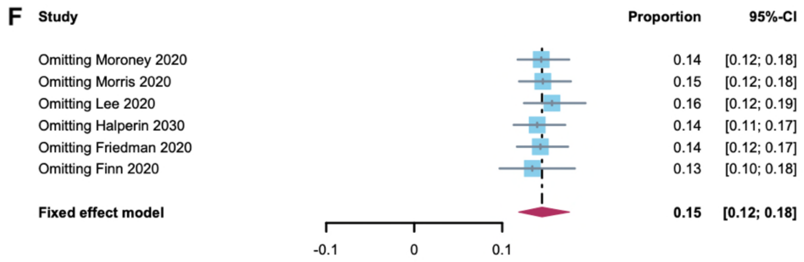
F
Study
Proportion
95%-CI
Omitting Moroney 2020
0.14
[0.12; 0.18]
Omitting Morris 2020
0.15
[0.12; 0.18]
Omitting Lee 2020
0.16
[0.12; 0.19]
Omitting Halperin 2030
0.14
[0.11; 0.17]
Omitting Friedman 2020
0.14
[0.12; 0.17]
Omitting Finn 2020
0.13
[0.10; 0.18]
Fixed effect model
0.15
[0.12; 0.18]
-0.1
0
0.1

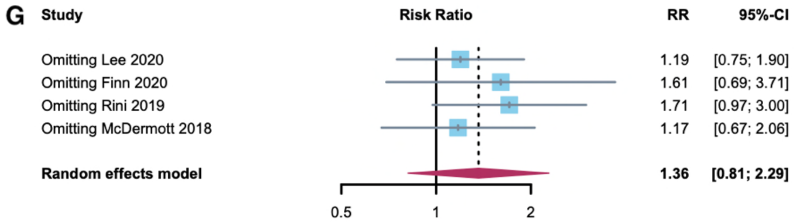
G
Study
Risk Ratio
RR
95%-CI
Omitting Lee 2020
1.19
[0.75; 1.90]
Omitting Finn 2020
1.61
[0.69; 3.71]
Omitting Rini 2019
1.71
[0.97; 3.00]
Omitting McDermott 2018
1.17
[0.67; 2.06]
Random effects model
1.36
[0.81; 2.29]
0.5
1
2

Supplementary figure 2. Forest plot for subgroup analyses. A. relative risk of atezolizumab plus bevacizumab-associated hypertension versus atezolizumab from the two randomized controlled trials; B. relative risk of atezolizumab plus bevacizumab-associated hypertension versus anti-VEGF agents from the three randomized controlled trials.

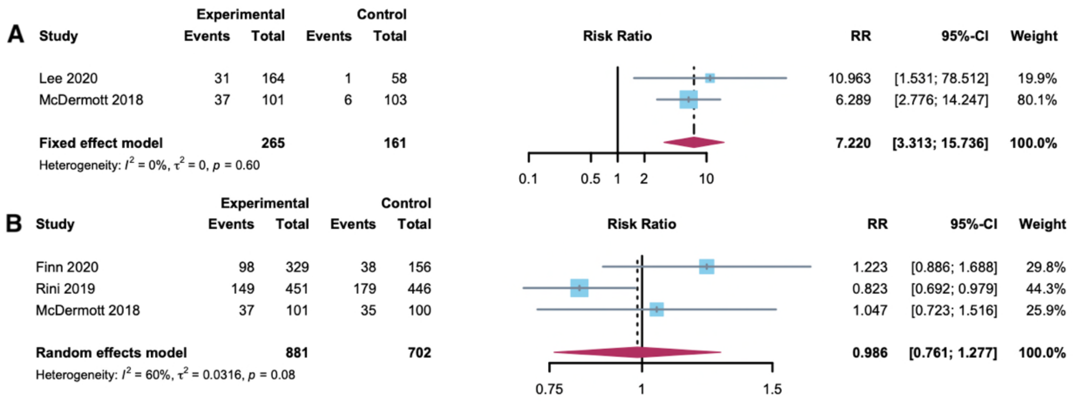

Supplementary figure 3. Funnel plot for publication bias. A. incidences of all-grade hypertension; B. incidences of high-grade hypertension; C. incidences of all-grade hypertension with RCC; D. incidences of high-grade hypertension with RCC; E. incidences of all-grade hypertension with non-RCC; F. incidences of high-grade hypertension with non-RCC; G. relative risk of atezolizumab plus bevacizumab-associated hypertension versus control from the four randomized controlled trials of patients.

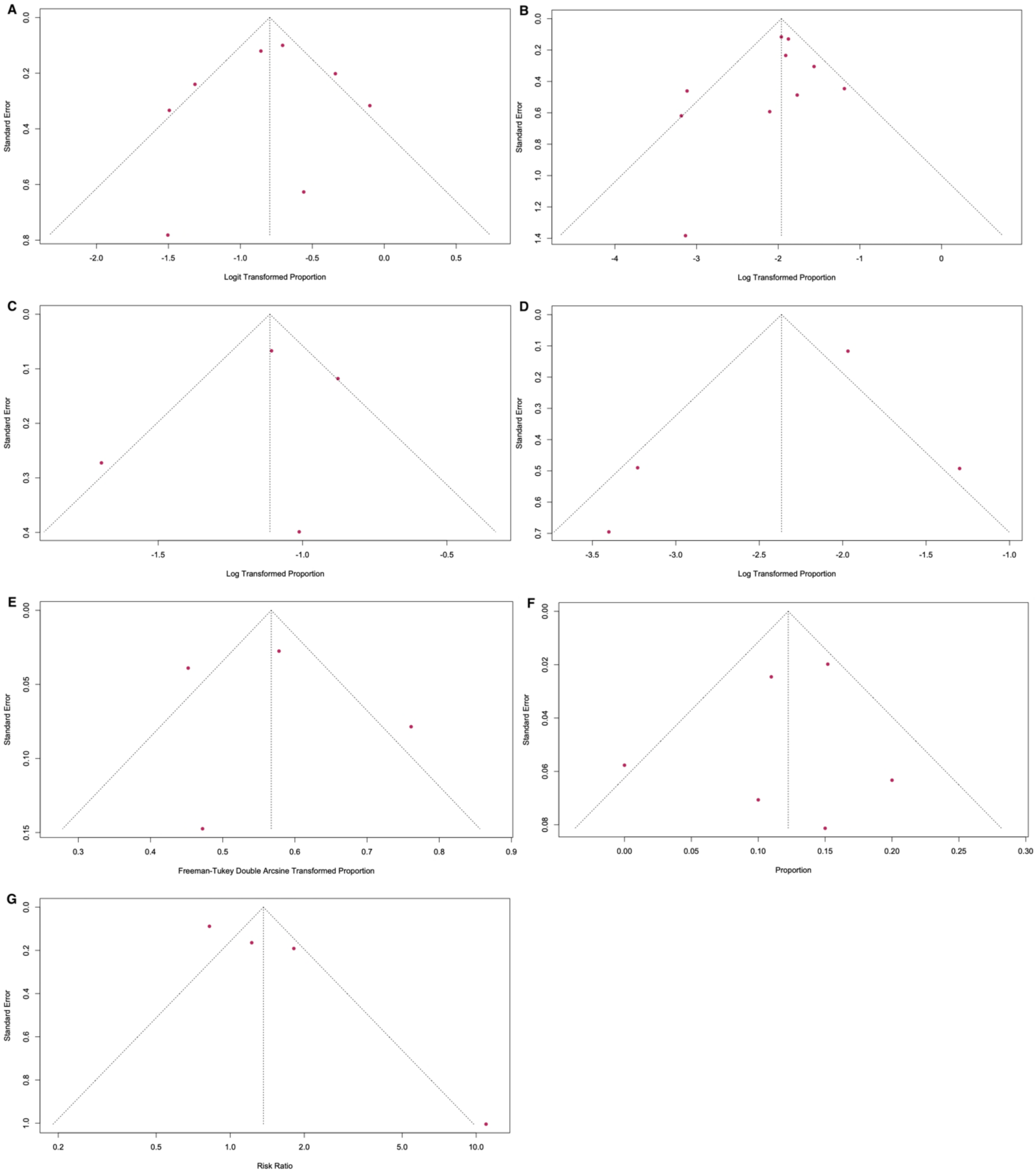

Supplementary figure 4. Assessment of publication bias by Egger's test. A. incidences of all-grade hypertension; B. incidences of high-grade hypertension; C. incidences of all-grade hypertension with RCC; D. incidences of high-grade hypertension with RCC; E. incidences of all-grade hypertension with non-RCC; F. incidences of high-grade hypertension with non-RCC; G. relative risk of atezolizumab plus bevacizumab-associated hypertension versus control from the four randomized controlled trials of patients.

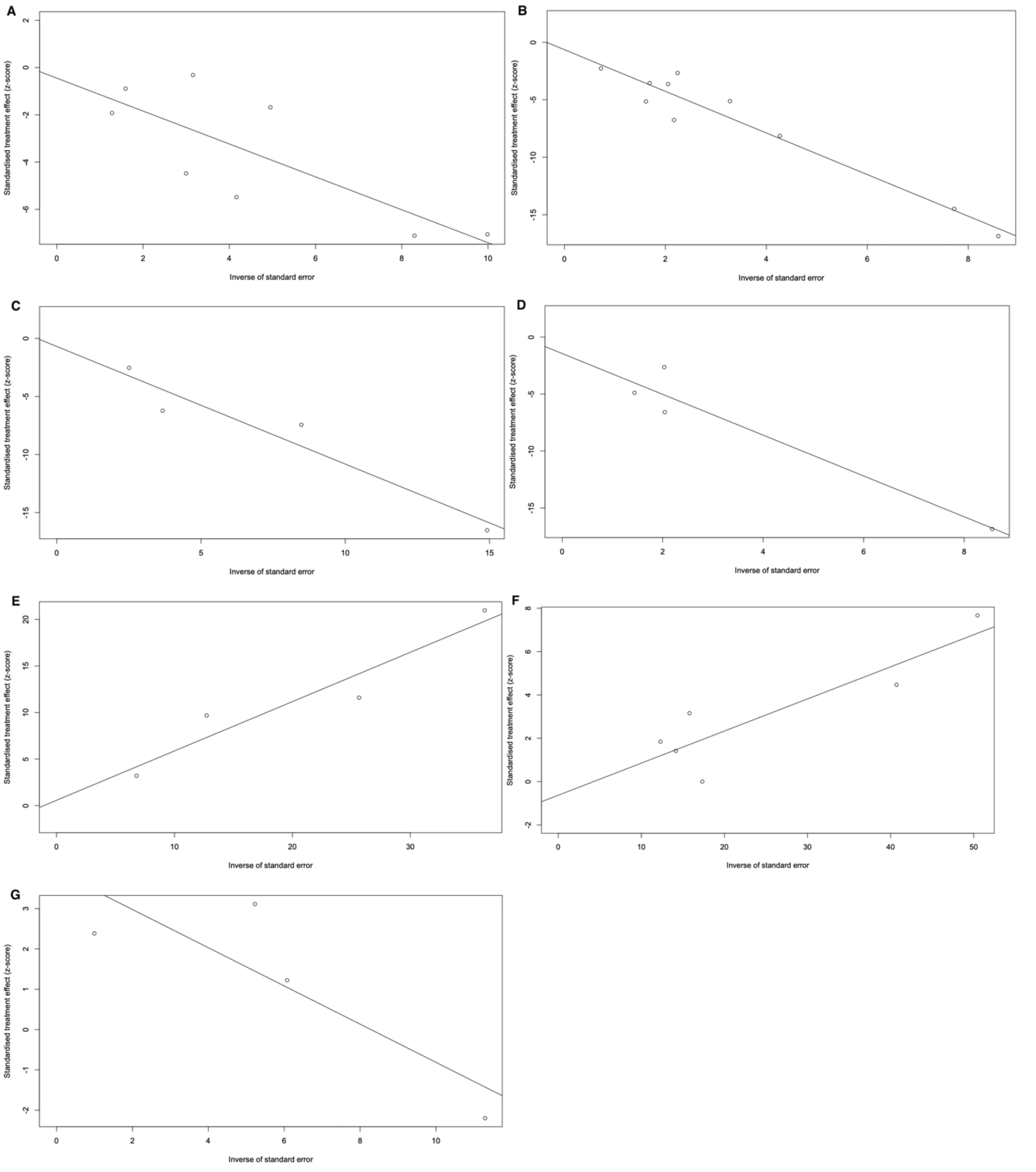

Supplementary figure 5. Assessment of publication bias by Begg's test. A. incidences of all-grade hypertension; B. incidences of high-grade hypertension; C. incidences of all-grade hypertension with RCC; D. incidences of high-grade hypertension with RCC; E. incidences of all-grade hypertension with non-RCC; F. incidences of high-grade hypertension with non-RCC; G. relative risk of atezolizumab plus bevacizumab-associated hypertension versus control from the four randomized controlled trials of patients.

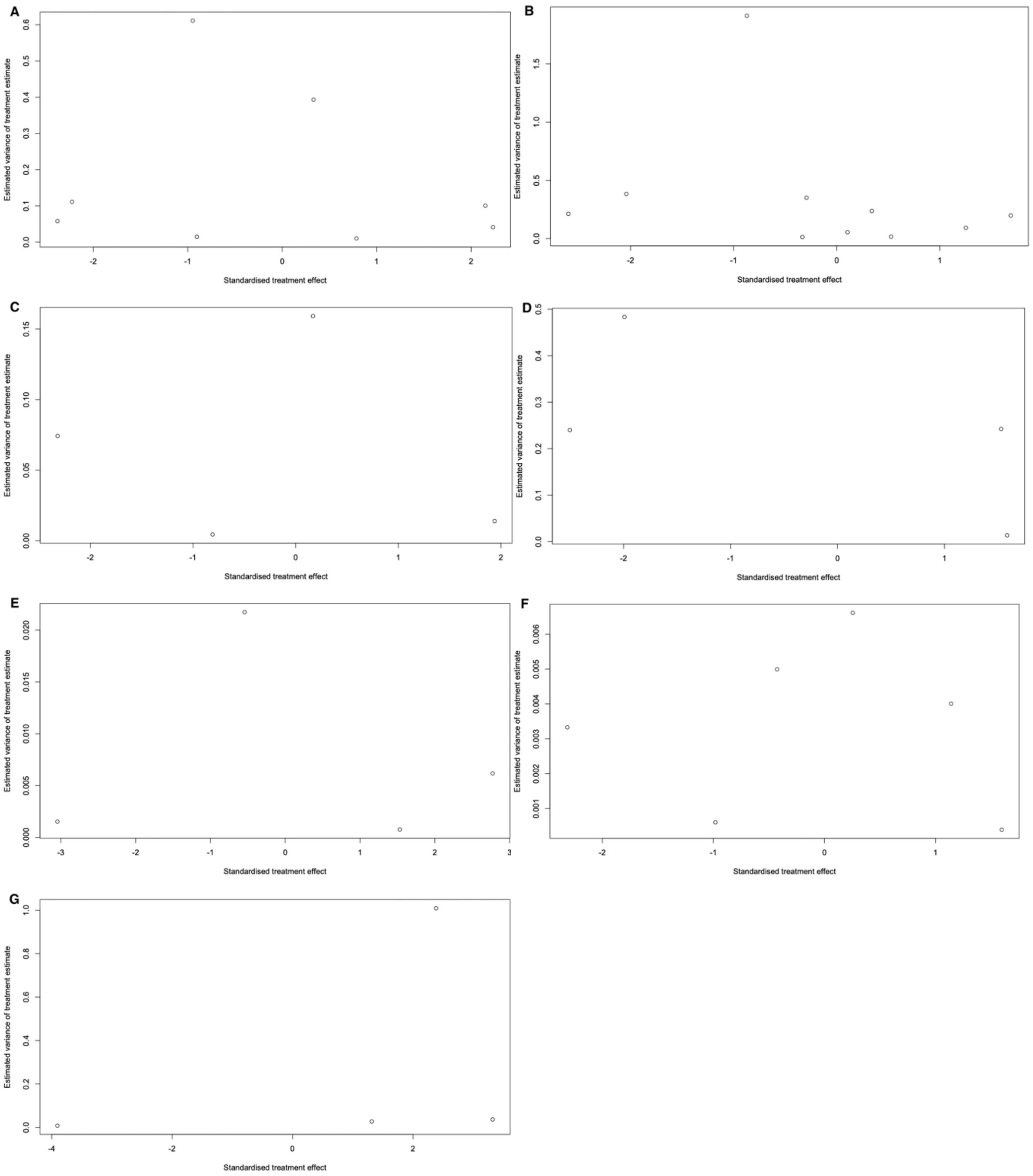

Supplement: Supplementary file 1 [file DataSheet_1.pdf]
